# Raman spectra of the GFP-like fluorescent proteins

**DOI:** 10.1007/s41048-018-0072-0

**Published:** 2018-11-02

**Authors:** Ye Yuan, Dianbing Wang, Jibin Zhang, Ji Liu, Jian Chen, Xian-En Zhang

**Affiliations:** 1 National Laboratory of Biomacromolecules, CAS Center for Excellence in Biomacromolecules, Institute of Biophysics, Chinese Academy of Sciences, Beijing, 100101 China; 2 College of Life Science and Technology, Huazhong Agricultural University, Wuhan, 430070 China; 3 College of Life Science, Hubei University, Wuhan, 430070 China

**Keywords:** Raman spectra, Fluorescent protein, Chromophore

## Abstract

The objective of the study was to elucidate optical characteristics of the chromophore structures of fluorescent proteins. Raman spectra of commonly used GFP-like fluorescent proteins (FPs) with diverse emission wavelengths (green, yellow, cyan and red), including the enhanced homogenous FPs EGFP, EYFP, and ECFP (from jellyfish) as well as mNeptune (from sea anemone) were measured. High-quality Raman spectra were obtained and many marker bands for the chromophore of the FPs were identified via assignment of Raman spectra bands. We report the presence of a positive linear correlation between the Raman band shift of C_5_=C_6_ and the excitation energy of FPs, demonstrated by plotting absorption maxima (cm^−1^) against the position of the Raman band C_5_=C_6_ in EGFP, ECFP, EYFP, the anionic chromophore and the neutral chromophore. This study revealed new Raman features in the chromophores of the observed FPs, and may contribute to a deeper understanding of the optical properties of FPs.

## Introduction

The Raman spectrum provides a “fingerprint” of the vibration and rotation of molecules. The conformation and structure of biomacromolecules such as DNA, protein chains, membrane proteins and lipids, as well as other structural data related to such molecules can be obtained using Raman spectroscopy (Bunaciu *et al*. 2015; Carey 1982; Tu 1982; Tuma 2005; Xu 2005).

In recent years, the Raman spectra of fluorescent proteins (FPs) have attracted much attention due to the unique optical properties of FPs and their wider applicability in molecular and cellular imaging. Analysis of the Raman spectra of GFP and its mutants revealed that the ground-state structure of the anionic form of the chromophore may be heavily dependent on the chromophore environment Bell *et al*. 2000). Femtosecond-stimulated Raman spectroscopy showed that skeletal motions are related to proton transfers which makes GFP in the fluorescent form (Fang *et al*. 2009). In addition, the Raman spectra of the red fluorescent protein, eqFP611, from the sea anemone, *Entacmaea quadricolor*, revealed photoinduced cis–trans isomerization of the chromophore (Davey *et al*. 2006). Resonance and pre-resonance Raman spectra of the photochromic fluorescent protein, Dronpa, demonstrated enhanced Raman band selectively for the chromophore, thus yielding important information on the chromophore structure (Higashino *et al*. 2016).

Based on the above findings, our focus was directed at the relationship between emission wavelengths of FPs and their Raman spectrum characteristics. To clarify this relationship, we measured the Raman spectra of a group of commonly used GFP-like FPs with diverse emission wavelengths (green, yellow, cyan, and red), including the enhanced homogenous fluorescent proteins EGFP, EYFP, and ECFP (from jellyfish) to mNeptune (from anemone). It is felt that the results of this study may not only enrich the understanding of Raman spectra in relation to FPs, but also benefit efforts associated with the rational design and directed evolution of FPs for practical purposes.

## Results and Discussion

### Raman spectra of fluorescent proteins

**Fig. 1:**
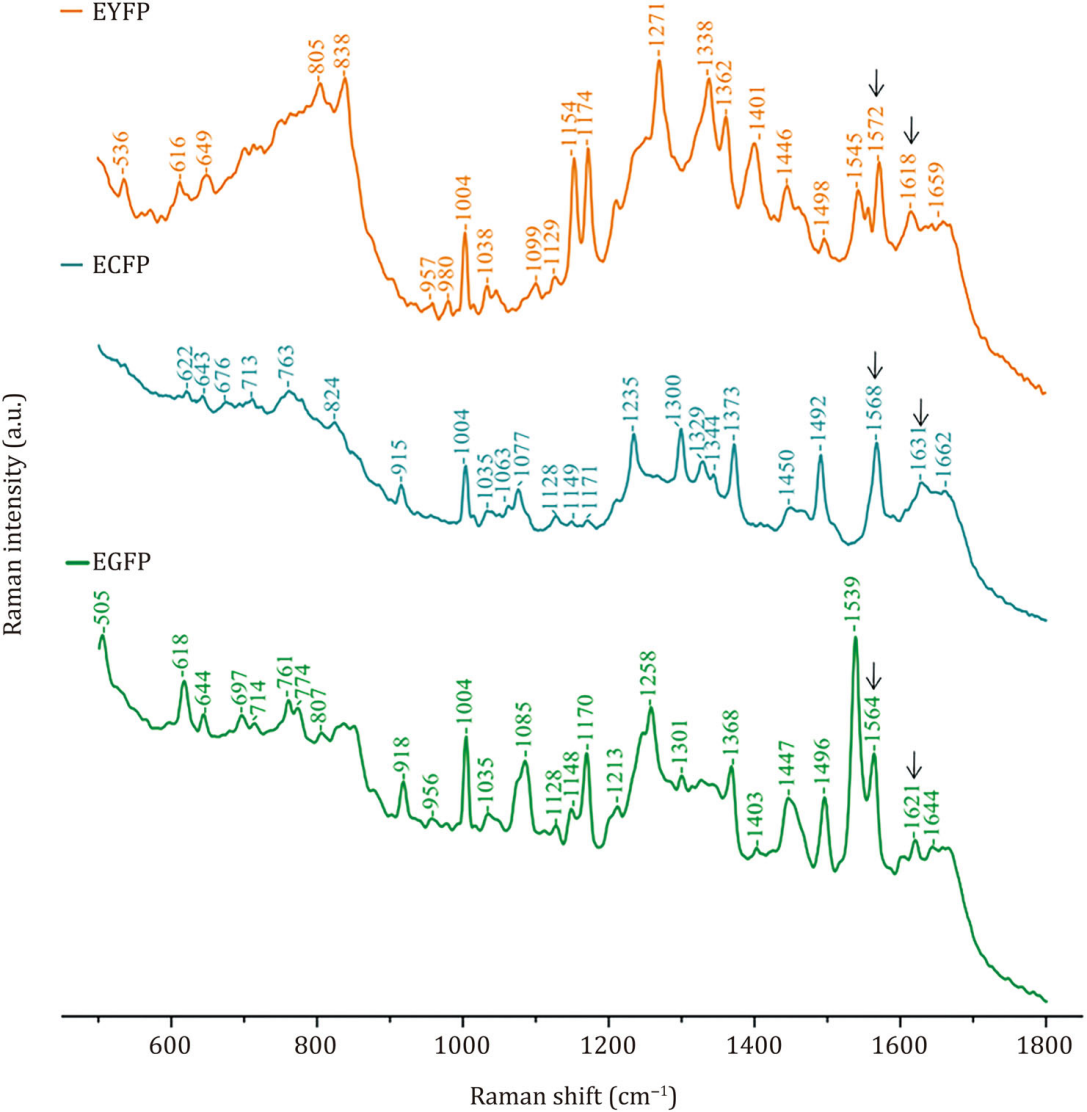
Raman spectra of EGFP, ECFP and EYFP

Raman spectra of EGFP, ECFP, and EYFP are shown (Fig. 1). Assignment of Raman bands for these FPs are presented (Table 1). Raman spectroscopy with 785-nm excitation was used to acquire the Raman spectra of FPs. This excitation wavelength selectively enhances the intensity of vibrational bands originating in the chromophore, and thereby avoids certain issues associated with strictly on-resonance Raman experiments such as fluorescence, photoisomerization, or sample degradation (Bell *et al*. 2000). As a result, most Raman spectra bands obtained in the study were produced by the chromophores of FPs, and only a few Raman spectra bands were due to the main chain groups and side chain groups on the β-barrel of fluorescent proteins. The Raman spectra bands of EGFP at 1664 cm^−1^, ECFP at 1662 cm^−1^ and EYFP at 1659 cm^−1^ are all assigned to Amide I modes (Table 1). The Raman spectra bands of EGFP at 1447 cm^−1^, ECFP at 1450 cm^−1^ and EYFP at 1446 cm^−1^ are assigned to side-chain CH_2_ group modes. The Raman spectra band at 1004 cm^−1^ is assigned to the aromatic side-chain mode of the FPs. The Raman spectra bands among 1220–1350 cm^−1^ were from Amide III.

**Table 1:** Raman shifts and mode assignments of EGFP, ECFP and EYFP

EGFP	ECFP	EYFP	Mode assignment
618	622	616	Ph^*a*^ C–H in-plane H bend
644	643		Ph C–H in-plane H bend
		649	–C=C str^*b*^
697			–N–H def^*c*^
807		805	–C–H out-of-plane def
		838	–C–H out-of-plane def
		980	–C–H in-plane H bend
1004	1004	1004	Aromatic side-chain mode of protein
1035	1035	1038	–C–H in-plane H bend
	1077		–C–H in-plane H bend
		1099	–C–H in-plane H bend
1128	1128	1129	–C–H in-plane H bend, Ph ring-H scissor
		1154	Ph C–H in-plane H bend, Bridge C6–H rock, imidazolinone ring, C12OH rock
1148	1149		–C–H in-plane H bend
1170	1171	1174	Ph ring-H bend, Bridge C6–H rock, Ph C–H def, imidazolinone ring def
1213			–C–H in-plane H bend
	1235		Ring combination bend-str
1258			–C–H in-plane H bend
		1271	–C–H bend
1301	1300		Ph ring-H bend
	1329		–C–N–H str
		1338	–C6–H rock
1368		1362	C–O–H def (O–H bend and C–O str, Ph hydroxyl group)
1403		1401	Ring combination bend-str
1447	1450	1446	Side chain CH2 group
1496	1492	1498	Ph ring str
1539			Imidazolinone + C=C str
		1545	–C–N–H str
1564	1568	1572	–C3=N1 str
1621	1631	1618	C5=C6 str
1644	1662	1659	–C4=O13 str

^*a*^Ph: Phenol

^*b*^str: stretch

^*c*^def: deformation

The chromophores of EGFP, EYFP, and mNeptune are mainly composed of the phenol group and the imidazolinone ring formed by propylene group bridging. As the phenol group consists of C, H, and O, the Raman spectra bands from the phenol group are due to the vibration of the phenol ring, C–H bonds of the phenol group, and the phenolic hydroxyl group. For example, the Raman spectra bands of EGFP at 618,1035, 1128 and 1170 cm^−1^, EYFP at 616,1038, 1129 and 1174 cm^−1^ and mNeptune at 621, 1107, 1119, 1156, and 1170 cm^−1^ are likely assigned to a C–H phenol bending mode. The bands of EGFP at 1496 cm^−1^, EYFP at 1498 cm^−1^ and mNeptune at 1480 cm^−1^ are assigned to a phenol ring stretching mode.

To configure the marker bands of the chromophore, the Raman spectra bands of ECFP were compared with those of EGFP, and EYFP. It was found that the band at 1368 cm^−1^ of EGFP and the band at 1362 cm^−1^ of EYFP were from stretching vibrations of the phenolic hydroxyl group on the side chain of 66Tyr in the chromophore. However, the bands of ECFP around 1368 cm^−1^ or 1362 cm^−1^ did not appear, due to the presence of an indolyl group, rather than a phenol group, on the side chain of 66Trp. Therefore, the band of ECFP at 1329 cm^−1^ due to the stretching vibration of indolyl C–N–H on the side chain of 66Trp, is likely a marker band of ECFP. In addition, there were other differences between the Raman spectra of EGFP, ECFP, and EYFP. For example, C_3_=N_1_ stretching of EGFP, ECFP, and EYFP each produced a Raman band at 1564, 1568 and 1572 cm^−1^, respectively; C_5_=C_6_ stretching of EGFP, ECFP, and EYFP each produced a Raman band at 1621, 1631 and 1618 cm^−1^, respectively; also, C_4_=O_13_ stretching of EGFP, ECFP, and EYFP each produced a Raman band at 1644, 1662 and 1659 cm^−1^, respectively. These results suggested that EGFP, EYFP, and ECFP may each have their respective featured Raman bands, and therefore may be distinguished by a comparison of their Raman spectra, although these enhanced FPs are highly homogenous. These features in the Raman spectra of FPs were strongly dependent on the environment, as well as the structure of the chromophore.

**Fig. 2:**
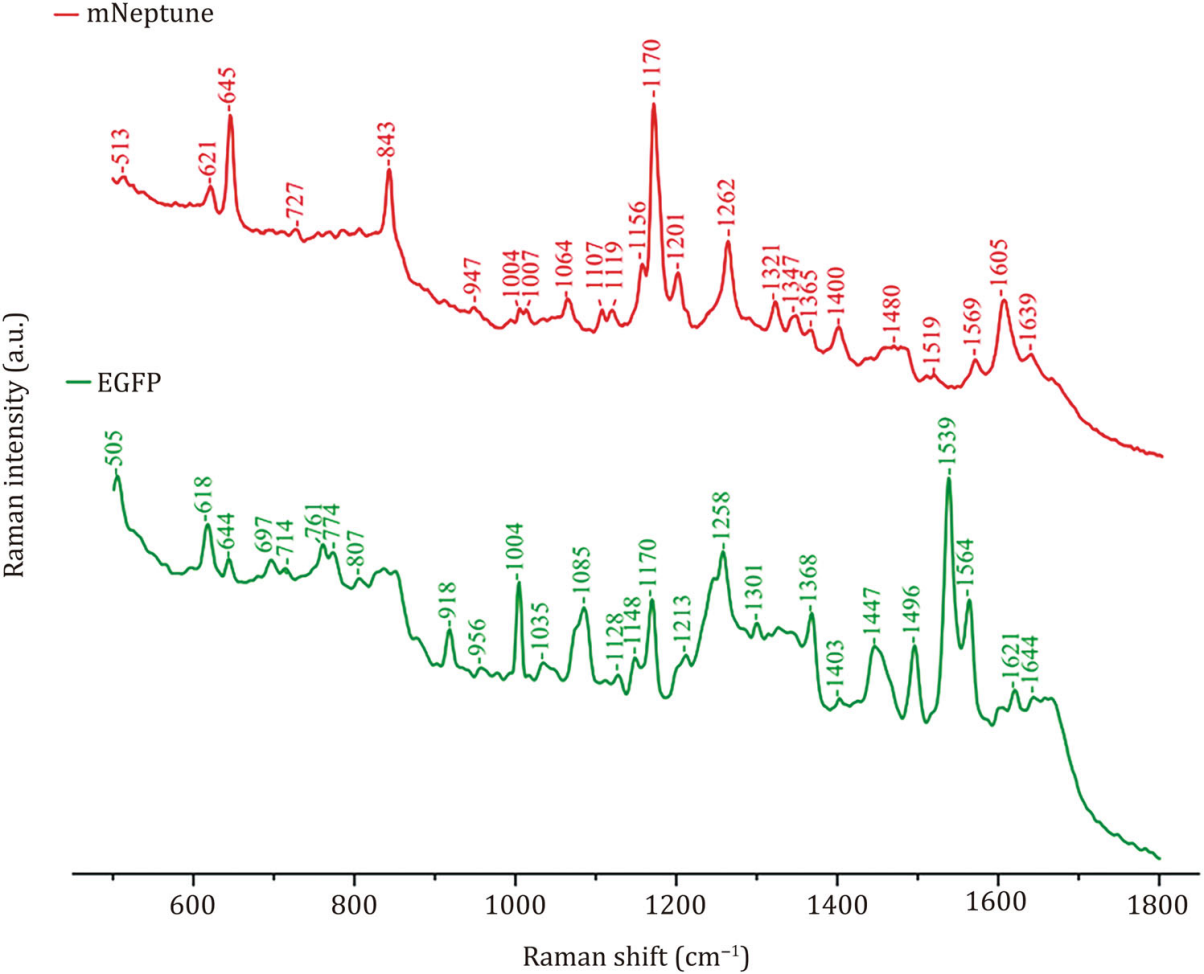
Raman spectra of mNeptune and EGFP

We also measured the Raman spectra of mNeptune, which is a GFP-like protein, originating in a sea anemone. As expected, its Raman spectrum was significantly different from that of EGFP, as the two FPs originated in different species (Fig. 2 and Table 2). Assignment of the Raman bands indicated that the bands of mNeptune at 1170, 1156 and 1201 cm^−1^ are marker bands, by which mNeptune may be distinguished from EGFP. Besides, mNeptune produced many Raman bands at 1320–1370 cm^−1^ which arose from the imidazolinone ring-related groups, whereas EGFP produced more bands at 1400–1500 cm^−1^ due to the presence of 66Tyr in its chromophore. We, therefore, hypothesized that different features in the Raman spectra of EGFP and mNeptune were mainly due to differences in their chromophore structures which resulted in obviously different molecular vibrations.

**Table 2:** Raman shifts and mode assignments of EGFP and mNeptune

EGFP	mNeptune	Mode assignment
618	621	Ph^*a*^ C–H in-plane H bend
644		Ph C–H in-plane H bend
697		–N–H def^*b*^
807		–C–H out-of-plane def
	645	CH=CH def cis in-phase wag
	843	–C–O skeletal str^*c*^
1004	1004	Aromatic side chain mode
1035		–C–H in-plane H bend
	1107	–C–H in-plane H bend
	1119	–C–H in-plane H bend
1128		–C–H in-plane H bend, Ph ring-H scissor
1148		–C–H in-plane H bend
	1156	Ph C–H in plane H bend, Bridge C_6_–H rock, imidazolinone ring def, C_12_OH rock
1170	1170	Ph ring-H bend(rock), Bridge C_6_–H rock, Ph C–H def;
	1201	–C–N str
1213		–C–H in-plane H bend
1258		–C–H in-plane H bend
	1262	Phenol C–H, Ph ring C_12_–O stretch, Ph ring-H rock
1301		Ph ring-H bend
	1321	–C–N str
	1347	–C–H rock
	1365	–C–O–H def^a^
1368		C–O–H def (O–H bend and C–O str, Ph hydroxyl group)
	1400	Ring combination bend-str;C_12_OH rock
1403		Ring combination bend-str
1447		Side chain CH_2_ group
	1480	Ph ring
1496		Ph ring str
1539		Imidazolinone + C=C str
1564		–C_3_=N_1_ str
	1569	Ring combination bend-str
	1605	C=C
1621		C_5_=C_6_ str
	1639	Amide I
1644		–C4=O_13_ str

^*a*^Ph: Phenol

^*b*^def: deformation

^*c*^str: stretch

### Linear correlation between the Raman band shift of C_5_=C_6_ in the chromophores and the excitation energy for FPs

**Fig. 3:**
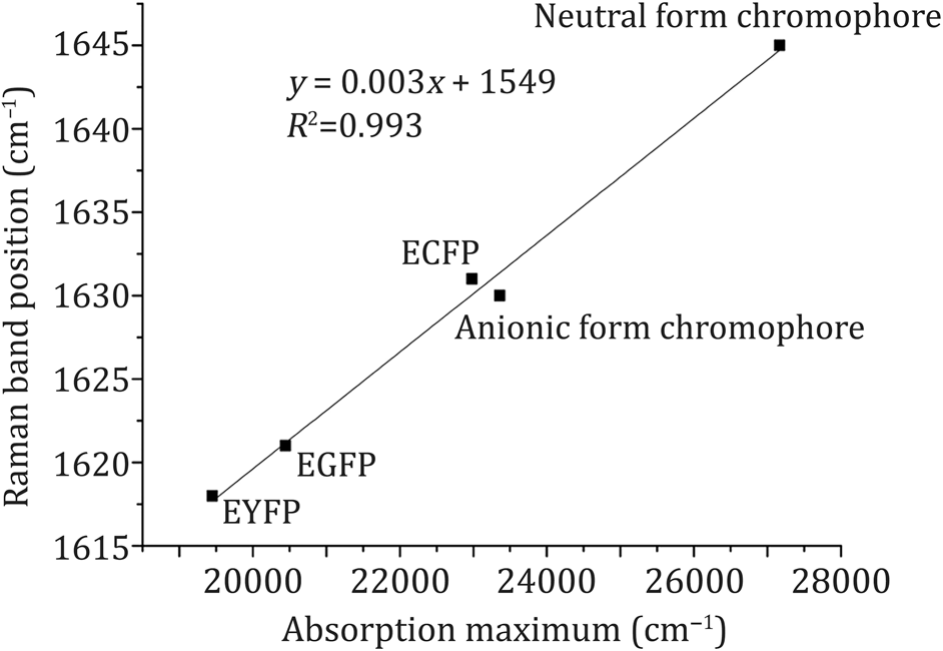
Plot of absorption maxima versus C_5_=C_6_ Raman band position for neutral form of chromophore, anionic form of chromophore, EGFP, ECFP and EYFP

The Raman band of C_5_=C_6_ in the chromophore is at 1621 cm^−1^ for EGFP, 1631 cm^−1^ for ECFP and 1618 cm^−1^ for EYFP (Tables 1, 3). A previous study illustrated that the Raman band of C_5_=C_6_ in the anionic chromophore form is at 1628 cm^−1^ and the band in the neutral chromophore form is at 1648 cm^−1^ (Bell *et al*. 2000). There was a positive linear correlation between the absorption maxima (cm^−1^) and the position of the Raman band of C_5_=C_6_ in EGFP, ECFP, EYFP, the anionic chromophore form, and the neutral chromophore form (Fig. 3).

**Table 3:** Comparison of characteristics of EGFP, ECFP and EYFP

FPs	Scheme of chromophore	*λ*_ex_/*λ*_em_^*a*^ (nm)	Raman bands		Raman spectra
			C_3_=N_1_	C_5_=C_6_	
EGFP		489/508	1564 cm^−1^	1621 cm^−1^	
ECFP		434/477	1568 cm^−1^	1631 cm^−1^	
EYFP		514/537	1572 cm^−1^	1618 cm^−1^	

^*a*^Excitation and emission maxima (Voityuk *et al*. 1998)

Excitation energy is dependent on the chromophore structure of the FP and its surrounding microenvironment, which is related to the absorption maximum. In the chromophore of EGFP, Tyr presents a conjugated ring with π electrons because of the connection of the imidazolidone ring and the phenolic group of 66Tyr by C_5_=C_6_. In this conjugated ring, the dihedral angle of C_4_–C_5_=C_6_–C_7_ is 177.67° (Arpino *et al*. 2012). As a comparison, the dihedral angle in ECFP is 173.38° (Lelimousin *et al*. 2009). Therefore, the π-conjugated plane in the chromophore of EGFP is larger than that of ECFP, illustrating lower excitation energy needed for EGFP. However, EYFP requires even lower excitation energy than EGFP, as π-stacking interaction between the chromophore and 203Tyr of EYFP leads to a more stable electronic state (Wachter *et al*. 1998). For these reasons, we postulate that the presence of a linear correlation demonstrates a direct relationship between the Raman band shift of C_5_=C_6_ and the excitation energy for FPs (Fig. 3). Obviously, the lower the excitation energy, the bigger the redshift of C_5_=C_6_ stretching mode, and vice versa.

### Analysis of the correlation between the Raman band shift of C_3_=N_1_ in chromophore and the photostability of FPs

**Fig. 4:**
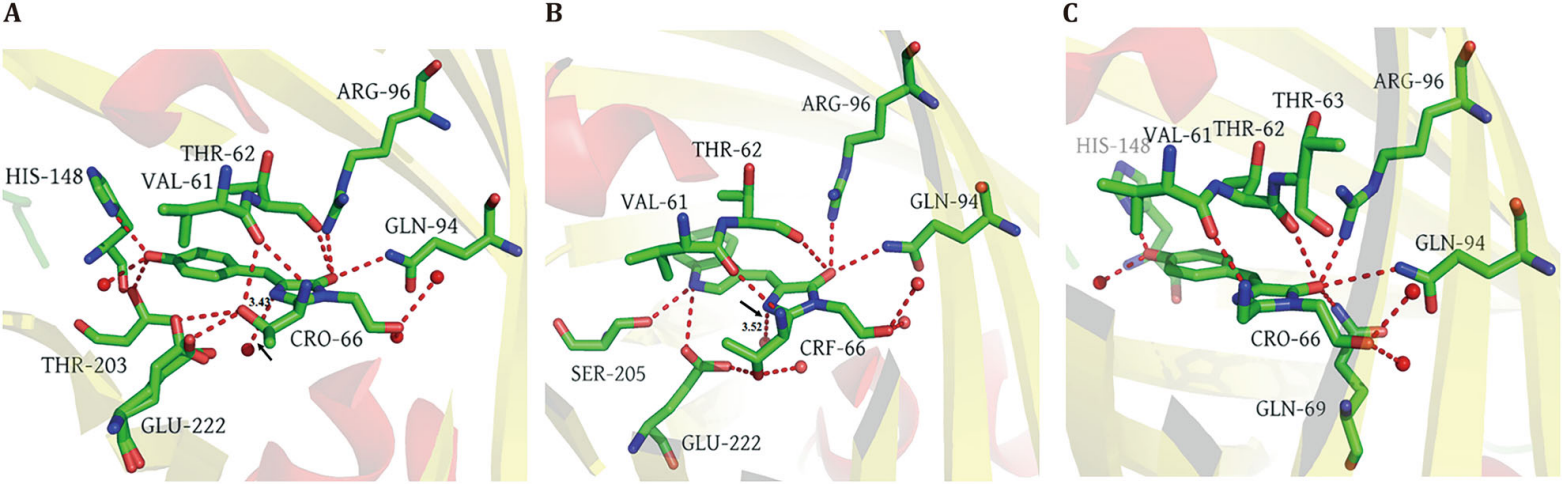
Chromophore hydrogen bond network of fluorescent proteins. *Red dashed lines* represent hydrogen bond. *Red spheres* denote H_2_O molecule. **A** Chromophore hydrogen bond network of EGFP (PDB Code:4EUL). **B** Chromophore hydrogen bond network of ECFP (PDB Code:2WSN). **C** Chromophore hydrogen bond network of EYFP (PDB Code:1YFP). *Black arrow* indicates the hydrogen bond between N1 and H_2_O molecule

The protonation of N_1_ in the chromophore of enhanced FPs plays an important role in chromophore stability, and exerts an effect on some optical properties of FPs (Wachter *et al*. 1998). We consider the interactions of N_1_ with its surrounding amino-acid residues and H_2_O may further stabilize the structure of the chromophore. Some evidence for this can be found in the Raman spectra of the FPs. For instance, the band at 1564 cm^−1^ in EGFP is a C_3_=N_1_ stretching mode (Table 3). However, the C_3_=N_1_ stretching mode is shifted to 1568 cm^−1^ in ECFP and to 1572 cm^−1^ in EYFP, respectively. It is evident from the chromophore hydrogen bond network of FPs that N_1_ and H_2_O form a hydrogen bond, through which H_2_O absorbs electrons from N_1_ (Fig. 4). This electron attraction effect causes a redshift of the C_3_=N_1_ mode, whereas such an effect is not observed in EYFP’s Raman spectrum, because EYFP lacks such a hydrogen bond. The length of the hydrogen bond in EGFP is 3.43 Å while it is 3.52 Å in ECFP, indicating a stronger attraction effect in EGFP compared to ECFP. Therefore, the C_3_=N_1_ mode in EGFP presents a bigger redshift compared to that of ECFP. Considering the fact that the photobleaching time ratio of ECFP to EGFP is 0.85, whereas the ratio of EYFP to EGFP is only 0.35 (Patterson *et al*. 2001), we propose that the redshift of the C_3_=N_1_ mode in the chromophore may possibly be related to the photostability of FPs. However, this contention may require further validation via experimental data.

## Conclusion

In summary, high-quality Raman spectra of a group of GFP-like FPs were obtained. Raman spectra of the FPs derived from GFP were evidently distinct from the RFP (mNeptune). Some marker bands were also found in the Raman spectra of GFP-derived FPs. These marker bands are mainly produced by their distinct chromophores. Among these bands, the Raman band shift of C_5_=C_6_ presents a positive linear correlation with the excitation energy for FPs. This study not only reveals new Raman features in the chromophore, but also illustrates the relationship between these features and the optical properties of FPs.

## Materials and Methods

### Reagents and materials

The vector pQE30, containing the gene clone of FPs, was purchased from Qiagen (Hilden, Germany). *E. coli* TG1 was used to express FPs. The primers used in this study are listed in Table 4.

**Table 4:** Primers used in this study

Primer name	Primer sequence(5′–3′)
EGFP-BamHI-F	ATATGGATCCATGGTGAGCAAGGGCGAGGA
EGFP-SacI-R	GAGCGAGCTCTTACTTGTACAGCTCGTCCAT
ECFP-BamHI-F	ATATGGATCCATGGTGAGCAAGGGCGAGGA
ECFP-SacI-R	GAGCGAGCTCTTACTTGTACAGCTCGTCCAT
EYFP-BamHI-F	ATATGGATCCATGGTGAGCAAGGGCGAG
EYFP-SacI-R	GCGTGAGCTCTTACTTGTACAGCTCGTCCAT
mNeptune-BamHI-F	ATAGGATCCATGGTGTCTAAGGGCGAAGAGCTGATTA
mNeptune-SacI-R	ATAGAGCTCTTACTTGTACAGCTCGTCCATGCCATTA

### Expression and purification of fluorescent proteins

The cDNAs of FPs were cloned into the BamH I and Sac I restriction sites of the pQE30 vector, using forward primers and reverse primers, respectively (Table 4). FPs were expressed in the *E. Coli* TG1 strain. Bacterial cultures were grown overnight in LB media containing 100 μg/mL ampicillin at 180 r/min at 37 °C, and further incubated at 25 °C for 12 h. The cells were harvested via centrifugation at 4500 r/min at 4 °C for 5 min, and the cell pellets were resuspended in binding buffer (20 mmol/L Tris–HCl, 500 mmol/L NaCl, 20 mmol/L imidazole, pH 8.0). Following cell lysis by French pressure (JNBIO, JN-02C, China), the FPs were purified using Ni–NTA His-Bind resin (GE Healthcare, USA) and a Superdex-200 size exclusion column (GE Healthcare, USA) according to the manufacturer’s instructions. Purified proteins were characterized using SDS–polyacrylamide electrophoresis. FPs were stored in Start Buffer (20 mmol/L Tris pH 7.9; NaCl 100 mmol/L) with concentration of 15–20 mg/mL for further analysis.

### Raman spectroscopy

For Raman measurements, 45 µl of protein solution was added into a sample cell and placed on the object stage. The sample was excited with a 785-nm pulsed laser at an excitation power of 50 mW/cm^2^, as 785-nm excitation allows probing of the chromophore site with minimal spectral interference from the surrounding protein environment.

The Raman spectra were acquired using Renishaw inVia Reflex confocal Raman microscope (Renishaw, UK) with a total collection time of 60 s for the recording of the spectra region from approximately 400–2400 cm^−1^. The laser beam was focused on the 200–300 µm point under the sample surface.

Raw data were processed by KnowItAll software, (Bio-Rad, USA) and Raman spectra were calculated by Savitzky–Golay smoothing. Raman spectra assignment was performed using KnowItAll software functional group database and references.
